# *Rhodotorula mucilaginosa* C2.5t1 Modulates Carotenoid Content and CAR Genes Transcript Levels to Counteract the Pro-Oxidant Effect of Hydrogen Peroxide

**DOI:** 10.3390/microorganisms7090316

**Published:** 2019-09-04

**Authors:** Sara Landolfo, Rossella Chessa, Giacomo Zara, Severino Zara, Marilena Budroni, Ilaria Mannazzu

**Affiliations:** Department of Agriculture, Università degli Studi di Sassari, Viale Italia 39, 07100 Sassari, Italy

**Keywords:** carotenoid, antioxidant response, hydrogen peroxide, CAR genes, *Rhodotorula mucilaginosa*

## Abstract

In order to contribute to the elucidation of the biological role of carotenoids, the cellular response to hydrogen peroxide was analyzed in the red yeast *R. mucilaginosa*. For that, the wild strain C2.5t1, that produces β-carotene, torulene, and torularhodin, and the albino mutant 200A6 that is incapable of producing detectable amounts of these carotenoids, were grown in the presence of increasing concentrations of hydrogen peroxide. In spite of the difference in carotenoid content, the two strains presented comparable resistance to the pro-oxidant that showed a minimum inhibitory concentration of 6 mM. When subject to 1 h treatment with 16 mM hydrogen peroxide the two strains increased catalase but not superoxide activity, suggesting that catalase plays a major role in cell protection in both the wild strain and the albino mutant. Moreover, C2.5t1 reduced its carotenoid content by about 40% upon hydrogen peroxide treatment. This reduction in carotenoids was in agreement with a significant decrease of the transcript levels of genes involved in carotenoid biosynthesis. Since an excess of β-carotene may enhance reactive oxygen species toxicity, these results suggest that C2.5t1 modulates carotenoid content to counteract the pro-oxidant effect of hydrogen peroxide.

## 1. Introduction

Carotenoids are the most common class of pigments occurring in nature. They constitute a group of more than 750 molecules responsible for the color of vegetables, microorganisms, and animals. Carotenoids are precursors of vitamin A [1] and different hormones [2], and show immunostimulating [3], photoprotective [2,4] and putative antitumor activities [5]. Moreover, they contribute to the pool of low molecular weight non-enzymatic compounds that detoxify oxidizing species, and therefore display antioxidant properties [1,6,7]. Accordingly, in recombinant strains of *Saccharomyces cerevisiae* carotenoids prevent the oxidative damage induced by reactive oxygen species (ROS) [8] and restore hydrogen peroxide resistance in cells unable to produce cytosolic catalase [9]. Thus, the antioxidant properties of carotenoids are largely accepted. Moreover, oxidative stress induced by hydroxytoluene butylate [10] and hydrogen peroxide [11] increases the production of β-carotene in red yeasts, and the inhibition of carotenoid biosynthesis determines an increase in antioxidant enzymes activity in *Rhodotorula mucilaginosa* [12]. However, it is also known that β-carotene may work as a pro-oxidant in the presence of high oxygen concentrations [13] and that β-carotene intracellular accumulation determines an increase in the sensitivity to ROS [14]. Moreover, β-carotene and other carotenoids induce ROS production, depolarize mitochondrial membrane in a dose dependent manner [15], and cause membrane stress [16]. In accordance with these evidences, a mitochondrial carotenoid oxygenase that degrades carotenoids, protects cells from oxidative stress [15]. Moreover, high carotenoid production results in increased expression of genes involved in pleiotropic drug resistance in recombinant strains of *S. cerevisiae*. Thus carotenoids, above a certain concentration, may become toxic for the cell and the induction of multidrug transporters may be necessary for the secretion of β-carotene [17].

Red yeasts ascribed to the species *R. mucilaginosa* are natural carotenoid producers [18,19] and represent an interesting biological model for the elucidation of the involvement of carotenoids in the cellular response to oxidative stress. Moreover, they have been proposed as cell factories for the production of unsaturated fatty acids [20], biosurfactants [21], other goods of biotechnological interest, and as biocontrol agents to counteract fungal spoilage on fruit [22,23]. Indeed, their biotechnological application may result in severe stress. They may undergo abiotic stress during biomass production. Moreover, they are subject to variations in environmental factors and to oxidative stress caused by the plant response to fungal attack, when used as biocontrol agents. However, in spite of this, little is known about the role of carotenoids in red yeasts response to ectopic pro-oxidants.

In this study, to gather further information on the biological role of carotenoids, the cellular response to hydrogen peroxide was evaluated in the wild strain of *R. mucilaginosa* C2.5t1 that produces β-carotene, torulene, and torularhodin, and in its albino mutant 200A6, incapable of producing detectable amounts of these carotenoids [19].

## 2. Materials and Methods

### 2.1. Yeast Strains and Culture Media

Strain C2.5t1, previously identified as *R. glutinis* and subsequently ascribed to *R. mucilaginosa* [24], is deposited at the DBVPG Industrial Yeast Collection (University of Perugia, Italy) with accession number DBVPG 10619. This yeast strain has already been thoroughly characterized for carotenoid production and subject to genomic and proteomic analyses [19,24,25,26]. Mutant 200A6 was obtained by UV mutagenesis of C2.5t1 [19]. This mutant, that does not produce detectable amounts of carotenoids and generates white colonies on YPGLY, is deposited at the Culture Collection of the Department of Agriculture, University of Sassari (Sassari, Italy). The medium used was YPGLY (yeast extract 1%, bacto peptone 2%, pure glycerol 8%, agar 2% when required). H_2_O_2_ was added to YEPGLY at the indicated concentrations. Yeasts were maintained on YEPD at 4 °C for short term conservation and on YEPD added with 20% glycerol at −80 °C for long term conservation.

### 2.2. Yeast Cultivation

Yeast cells were precultured in 20 mL of YPGLY and incubated at 30 °C, O/N under shacking conditions (180 rpm) and 10^6^ cells/mL of preculture were inoculated in 50 mL in 250 mL Erlenmeyer baffled flasks. Flasks were incubated as indicated above. Cell growth was monitored by evaluating either total cell count, dry weight of biomass, or viable cell count. For the determination of minimal inhibitory concentration, yeast cells were cultured in 24-well microplates (single well volume 1 mL) incubated in a Spectrostar Nano (BMG Labtech, Germany). The optical density at 600 nm (OD_600_) was evaluated every 15 min. Incubation was carried out at 30 °C. Before the OD_600_ measurement, the plate was subjected to 1 min linear shaking at 200 rpm. Data were mean ± sd of three independent replicates.

### 2.3. Carotenoid Extraction

Carotenoid extraction was carried out in petroleum ether [27] with slight modifications [19]. For the carotenoid analysis, UV-visible scanning spectra of petroleum ether extracts of carotenoids were recorded at λ ranging from 400 and 600 nm, as already described [19] using a SmartSpecTM plus spectrophotometer (BioRad, Milan, Italy). The total carotenoid concentration was expressed as β-carotene-equivalents with respect to a calibration curve obtained by utilizing pure β-carotene (Sigma Adrich, Milan, Italy) [19]. Data were obtained in triplicate from at least three biological replicates.

### 2.4. Biochemical Methods

Yeast crude extracts for enzymatic assay were prepared in 0.1 M Tris (pH 7.6) following a mechanical lysis protocol [28]. Crude extracts for zymogram and Western blotting were prepared in 50 mM potassium phosphate buffer (pH 7.0) containing PMSF (Sigma Aldrich, Milan, Italy) [29]. Total protein content was assayed according to Bradford [30]. The integrity of the crude extracts and the amount of proteins loaded in each lane were checked on SDS PAGE stained with Coomassie blue.

Catalase activity was determined as indicated by Luck [31] and expressed as units/mg of protein. Zymogram, for the determination of superoxide dismutase activity, was performed according to Gamero-Sandemetrio [32].

For the Western blotting, crude extract containing comparable amounts of proteins (15 μg) were loaded on polyacrilamyde gel (10%) and, after electrophoresis, proteins were transferred to PVDF membrane using a TransBlot SD semidry transfer cell (BioRad, Milan, Italy). Membranes were probed with anti-Sod2p (Enzo Life Sciences, Roma, Italy) at 1:3000 dilution as the first antibody and goat anti-rabbit IgG linked to alkaline phosphatase (Sigma Aldrich, Milan, Italy) at a 1:5000 dilution as the second antibody. Immunodetection was performed by the BCIP/NBT staining method (Sigma Aldrich, Milan, Italy).

### 2.5. RT-PCR

Total RNA extraction, retrotranscription, and qPCR amplification conditions were as described [26]. Primers used are reported in Table 1. For accurate quantification of RT-PCR products, at least three technical replicates of five biological replicates were carried out. Relative expression was evaluated with respect to *ACT*1 used as a housekeeping gene. The GenBank accession numbers for the CDS sequences of *HMG*1, *ERG*12, *CAR*2, *CAR*1, *CAR*0, and *ACT*1 of *R. mucilaginosa* C2.5t1 are KY937962, KY937963, KY937964, KY937966, KY937965, and KY937967, respectively [26].

### 2.6. Data Analysis

Unless otherwise stated, experiments were carried out in triplicate from independent precultures. The statistical analyses of the data were performed using ANOVA followed by Tukey Kramer HSD test (all pair comparison) using the JMP version 3.1.5 software (SAS Institute Inc., Cary, NC, USA). Statistical differences among gene expression levels in different samples were assessed by using the nonparametric Wilcoxon two group test (*p* < 0.05) by means of the statistical environment R (R Core Team, 2018) and the package “pcr” [33].

## 3. Results and Discussion

**Figure 1:**
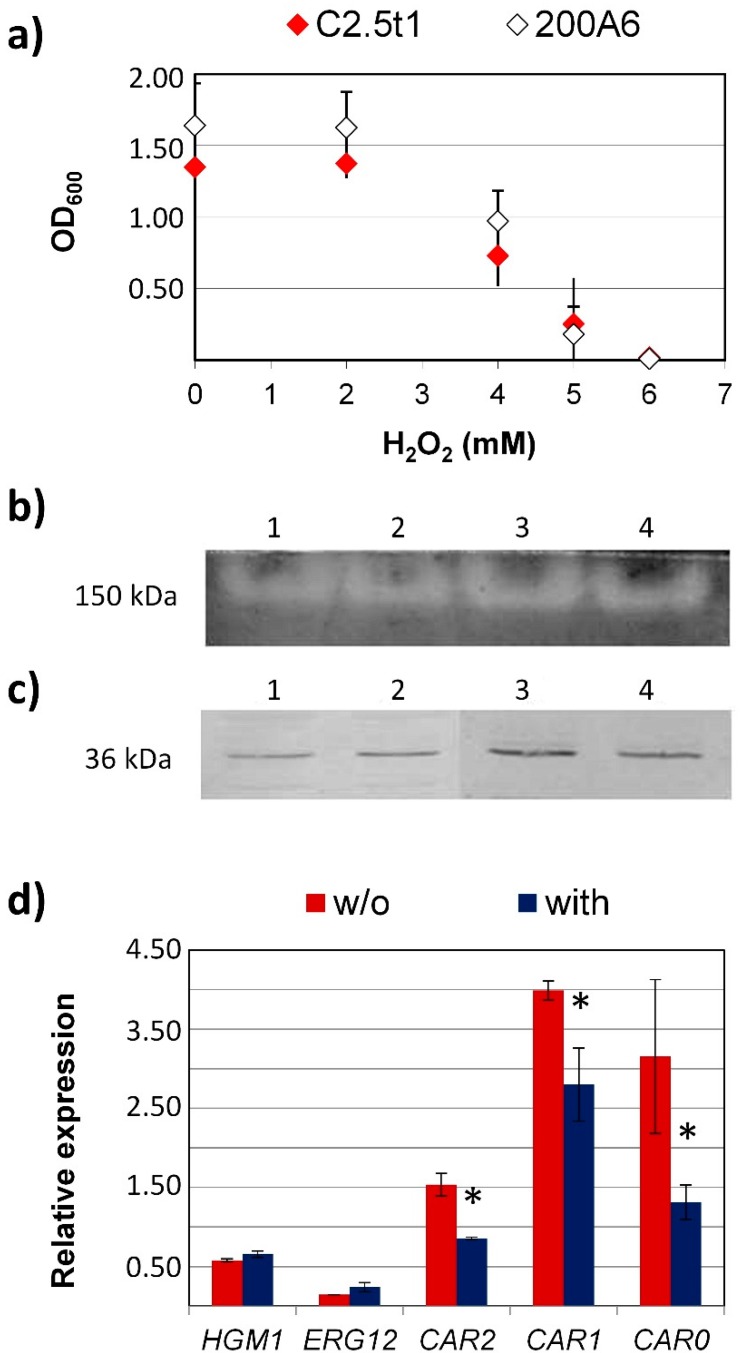
Investigating *R. mucilaginosa* cellular response to H_2_O_2._ (**a**) Hydrogen peroxide minimal inhibitory concentration after 48 h growth in YEPGLY with increasing concentration of H_2_O_2_ (0–7 mM), (**b**) zymogram, and (**c**) Western blot of superoxide dismutase after 1 h treatment in YEPGLY without and with 16 mM H_2_O_2_. Lanes 1–4: C2.5t1 without H_2_O_2_, C2.5t1 with H_2_O_2_, 200A6 without H_2_O_2_, and 200A6 with H_2_O_2_. (**d**) C2.5t1 transcript levels of carotenogenic genes after 1 h treatment in YEPGLY without and with 16 mM H_2_O_2_. Relative expression was evaluated with respect to actin used as a housekeeping gene. All data are means ± standard deviations of three technical replicates of three independent experiments. * indicates significant differences among means (*p ≤* 0.05) as determined by the nonparametric Wilcoxon two group test (*p* < 0.05).

To evaluate whether carotenoids are involved in the cellular response to hydrogen peroxide, about 1 × 10^6^ cell/mL of the wild strain of *R. mucilaginosa* C2.5t1 that produces β-carotene, torulene, and torularhodin, and of its albino mutant 200A6 which is incapable of producing detectable amounts of these carotenoids, were inoculated in liquid YPGLY and grown with increasing concentrations of hydrogen peroxide (from 0 to 7 mM H_2_O_2_) for up to 48 h. In spite of the difference in carotenoid content, the two strains showed comparable behavior. Both strains proved capable of growing in the presence of up to 5 mM H_2_O_2_, while no growth was observed at 6 mM (Figure 1a).

To gather further information on *R. mucilaginosa* cellular response to hydrogen peroxide, the two strains were subjected to H_2_O_2_ treatment. To do that, C2.5t1 and 200A6 were grown in 50 mL liquid YEPGLY in 250 mL baffled flasks. After 24 h at 30 °C under shaking conditions (180 rpm) cells were harvested, washed, and about 1 × 10^6^ cell/mL were resuspended in YEPGLY without (control) and with 16 mM H_2_O_2_ and incubated for 1 h, as stated above. At this concentration, hydrogen peroxide, although impairing cell growth, had no effect on viability as shown by viable plate counts (1 × 10^6^ UFC/mL and 1.3 × 10^6^ UFC/mL in C2.5t1 and 200A6, respectively). In parallel, cell samples were analyzed by flow cytometry after propidium iodide (PI) staining [29]. The PI penetrates the cells with damaged cell membranes, and flow cytometry of PI stained cells, besides gathering information on cell membranes permeability, provides an indirect measure of cell viability [29]. Results obtained (Figure S1) showed no differences between control and treated cells, thus, indicating that 1 h incubation with 16 mM hydrogen peroxide has no effect on viability and cell membrane permeability.

The involvement of the antioxidant enzymes, superoxide dismutase and catalase, in the cellular response to hydrogen peroxide was, therefore, evaluated in the two strains. Superoxide dismutase (SOD) and catalase are two ROS enzymatic scavengers. However, while catalase breaks H_2_O_2_ into water and oxygen, and is, therefore, directly involved in H_2_O_2_ detoxification, superoxide dismutase is devoted to the detoxification of superoxide anion, although yeast cells may significantly increase SOD activity after hydrogen peroxide treatment [34].

Since crude extracts of carotenogenic yeasts are pigmented, and are, therefore, not suitable for the evaluation of superoxide dismutase enzymatic activity through the nitroblue tetrazolium reduction test [35], SOD expression level was evaluated by Western blot using an antiSod2p polyclonal antibody. A band of about 36 kDa, compatible with the size of a MnSOD already reported in *Rhodosporidium toruloides* (UniProtKB, A0A0K3CNV9) was observed (Figure 1b). To further confirm this result, crude extracts of C2.5t1 and 200A6 were also analyzed by zymogram technique for SOD detection, validated by Gamero Sandemetrio et al. [32], as a quantitative assay for Sod2p quantification on gel. This technique highlighted the presence of a unique band of approximately 150 kDa, which was compatible with the production of the homo-tetrameric form of Sod2p in non-denaturing gel (Figure 1c). Images of Western blot membranes and zymograms were acquired with a Chemidoc XRS (BioRad, Milan, Italy). Densitometric analysis carried out with Quantity One software (BioRad, Milan, Italy) revealed no significant variations (*p* < 0.05) in the amount of the target protein in cells sampled after 1 h in YEPGLY without and with H_2_O_2_, thus suggesting that Sod2p may not be involved in the antioxidant cell response this pro-oxidant.

Catalase activity, evaluated as indicated by Luck [31], was comparable in the two strains when incubated in YEPGLY without H_2_O_2_ (22.53 ± 1.5 and 26.01 ± 2.98 U/mg of protein in C2.5t1 and 200A6, respectively). Catalase activity increased significantly (*p* < 0.05) after 1 h hydrogen peroxide treatment reaching 42.02 ± 5.4 and 37.16 ± 2.55 U/mg of protein in C2.5t1 and 200A6, respectively. Thus, catalase plays a role in the antioxidant response to hydrogen peroxide, no matter the carotenoid content.

The two strain were subject to carotenoid extraction and quantification as already described [19] (Figure S2a). The albino mutant 200A6 confirmed the complete lack of carotenoids under all conditions tested (data not shown). C2.5t1, in contrast with that observed in other fungi that increase the content of β-carotene in the presence of H_2_O_2_ [36,37,38], reduced significantly (*p* < 0.05) the intracellular content of carotenoids upon 1 h H_2_O_2_ treatment (0.96 ± 0.24 mg/L in the control and 0.36 ± 0.07 mg/L after 1 h treatment with H_2_O_2_, (Figure S2b).

To further investigate the reason why hydrogen peroxide administration was accompanied by a reduction in carotenoid content, the transcript levels of carotenogenic genes were evaluated in C2.5t1. For that, total RNA was extracted, retrotranscribed, and the transcript levels of the genes of interest were evaluated by qPCR as already described [26]. In particular, the molecular targets considered were *HMG*1, *ERG*12, *CAR*2, and *CAR*1 coding for 3-hydroxymethyl 3-glutharyl CoA reductase, mevalonate kinase, phytoene synthase/lycopene cyclase, and phytoene dehydrogenase, respectively [26]. *HMG*1 and *ERG*12 are involved in the mevalonate pathway. Hmg1p is a key regulator of isoprenoid biosynthesis and Erg12p is involved in the biosynthesis of isopentenyl pyrophosphate (IPP), an intermediate in the biosynthesis of terpenoids [26]. Although not exclusively involved in carotenogenesis, they undergo an increase in their transcript levels during carotenoid biosynthesis and play a role in this biosynthetic pathway [39,40]. *CAR*2 and *CAR*1 are involved in the late carotenogenic pathway [26]. In particular, Car2p is a bifunctional enzyme implicated in the biosynthesis of phytoene and the cyclisation of lycopene, while Car1p catalyzes the sequential desaturation of phytoene. In *R. mucilaginosa,* the transcript levels of these genes are rather low during carotenogenesis in accordance with the low expression of carotenogenic proteins [25,26]. The *CAR*0 gene, coding for a putative carotenoid dioxygenase, was also considered [26]. Carotenoid dioxygenases are involved in the selective oxidative cleavage of carotenoids to produce apocarotenoids [41] and previous results have shown that *CAR*0 gene transcript levels increase with the increase in carotenoid content in *R. mucilaginosa* C2.5t1 [26].

As shown in Figure 1d, C2.5t1 showed no significant variations in the relative expression of *HMG*1 and *ERG*12 genes, thus suggesting that hydrogen peroxide has no effect on the carbon flux through the mevalonate pathway. Interestingly, in accordance with the significant decrease in carotenoid content, C2.5t1 decreased the transcript levels of *CAR*2 and *CAR*1 genes upon H_2_O_2_ treatment. Moreover, the *CAR*0 gene transcript level also decreased following H_2_O_2_ treatment. Thus, although a bleaching effect of hydrogen peroxide on carotenoids cannot be excluded, also the downregulation of carotenogenic genes seems to contribute to the reduction in total carotenoid content.

## 4. Conclusions

The results presented indicate that the lack of detectable amounts of β-carotene, torulene, and torularhodin does not affect the minimal inhibitory concentration of H_2_O_2_ that, in the albino mutant 200A6, is comparable with that of the parental strain C2.5t1. Thus, in spite of their antioxidant role [37,42], carotenoids do not seem to contribute to hydrogen peroxide resistance in *R. mucilaginosa* C2.5t1. On the contrary, the significant increase in catalase activity upon H_2_O_2_ treatment indicates that this enzyme plays a major role in cell protection from ROS both in the presence and in the absence of carotenoids.

Indeed, the antioxidant role of carotenoids depends on their amount and qualitative composition and an excess of β-carotene may result in an increase in ROS sensitivity [14]. In accordance with these findings, the wild strain C2.5t1 reduced its carotenoid content and the transcript levels of carotenogenic genes following 16 mM hydrogen peroxide treatment. These results are compatible with the hypothesis that C2.5t1 modulates carotenoid content to counteract the pro-oxidant effect of hydrogen peroxide.

## Figures and Tables

**Table 1:** Primers used for q-RT PCR.

Molecular Target	Primer Name	Sequence
*HMG1*	*HMG1*F	5′-TCACGCTCCACTCGCTCAAC-3′
	*HMG1*R	5′-CGAGGACAAGATGGGGTTGG-3′
*ERG12*	*ERG12*F	5′-CAGTCGGCGCAGGCGTTCTT-3′
	*ERG12*R	5′-GGACGCCGTGCGAGTAGAGC-3′
*CAR2*	*CAR2*F	5′-CCTTCCTCGCCAACGCCTCT-3′
	*CAR2*R	5′-CGTTGTTGGCGTACAGGAGG-3′
*CAR1*	*CAR1*F	5′-CGGTCCCTCGCTCTACCTCA-3′
	*CAR1*R	5′-CCTTGTCCGGGAAGACGATG-3′
*CAR0*	*CAR0*F	5′-CCGTCGGGTACTACAGTCTC-3′
	*CAR0*R	5′-TAGCTCAGATATGGCGGCAA-3′
*ACT1*	*ACT1A*F	5′-CGTTCAGATCCAGGCCGTCT-3′
	*ACT1A*R	5′-CGGCAATGCAAACCCTTCAT-3′

## Supplementary Materials

The following are available online at https://www.mdpi.com/2076-2607/7/9/316/s1.
