# Supplementary material for: Rhodotorula mucilaginosa C2.5t1 Modulates Carotenoid Content and CAR Genes Transcript Levels to Counteract the Pro-Oxidant Effect of Hydrogen Peroxide
Source: Microorganisms. 2019 Sep 4;7(9):316. doi: 10.3390/microorganisms7090316 (PMC6780508; doi:10.3390/microorganisms7090316)
Supplement: Supplementary file 1 [file microorganisms-07-00316-s001.pdf]

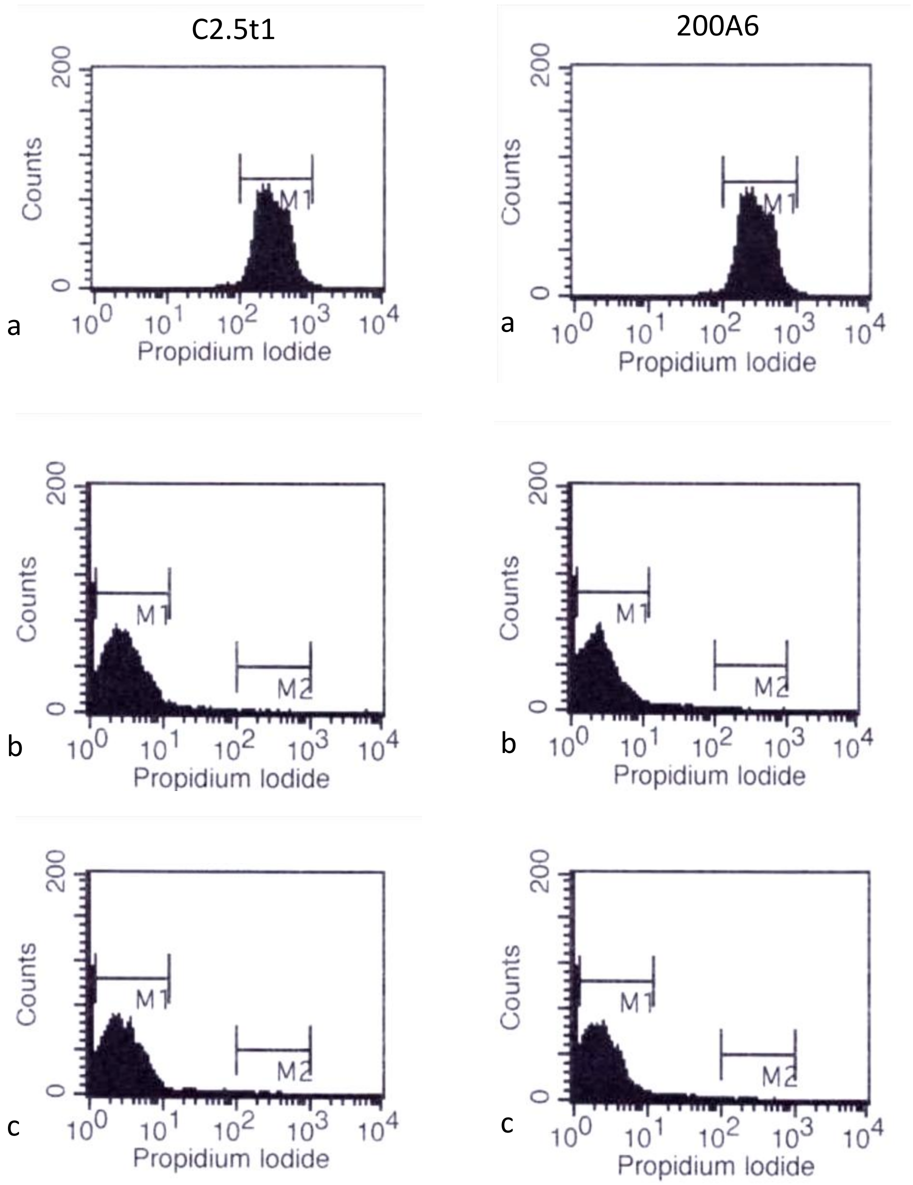

Supplemental Figure 1. Flow cytometry of PI stained cells. Cells samples were re-suspended in PBS (NaCl 8.0 g /l, KCl 0.20 g/l, Na_2HPO_4 1.44 g/l, KH_2PO_4 0.24 g/l, pH 7.4) to a final concentration of $1*10^6$ cells/ml. Cell suspensions were stained as follows: 10 µl PI stock solution (10 mg/ml) in PBS was added to 1 ml of cell suspension just prior to the analysis. Samples were analyzed with a BD FACSCalibur equipped with argon laser (15 mW, emission 488 nm) for the aquisition of fluorescence: FL1 (green, 525 nm), FL2 (yellow, 575 nm) and FL3 (orange-red, 620 nm). Fluorescence was detected in fluorescence channel 3. a) Cells treated with 70% ethanol (positive control of cell membrane permeability); b and c) Cells sampled after 1 hour incubation in YEPGLY w/o and YEPGLY with 16 mM H_2O_2, respectively.

a

b

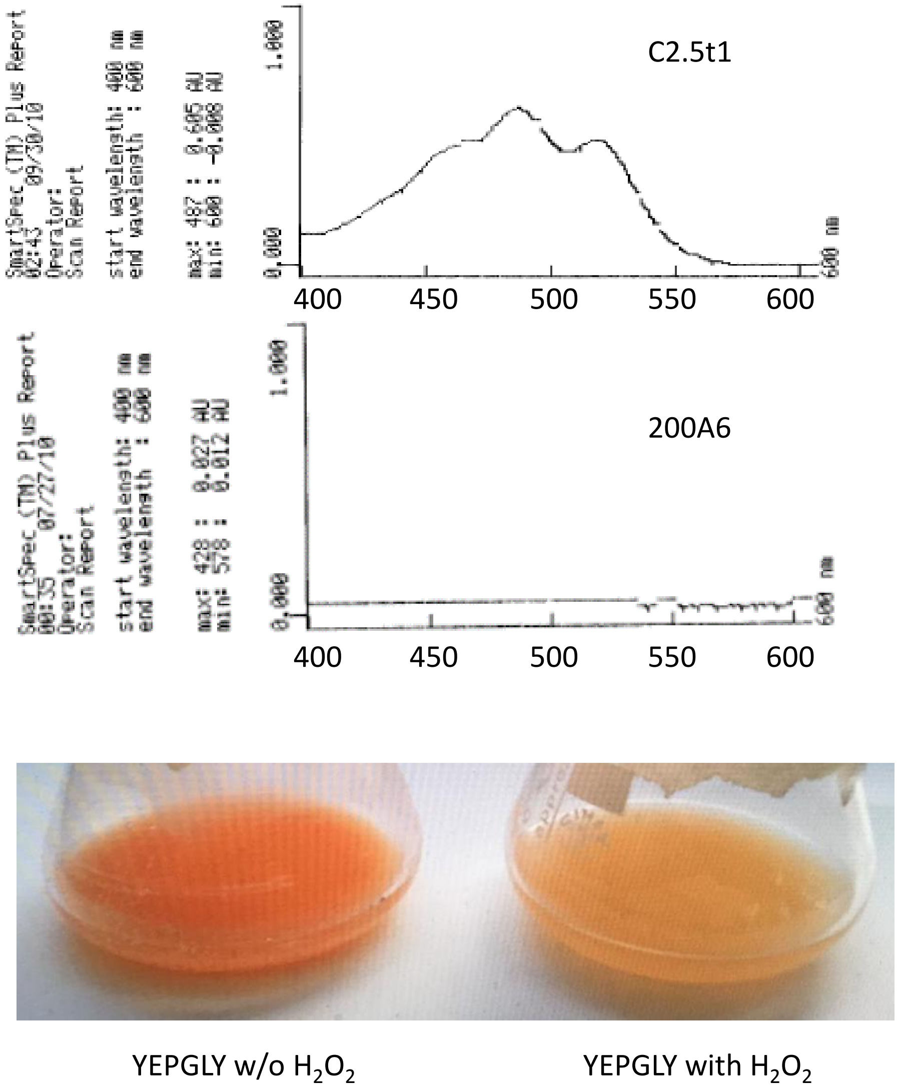

Supplemental Figure 2. a) Carotenoids quantification. Representative images of UV/VIS scanning spectra of total carotenoids. B) Effect of hydrogen peroxide on carotenoids content in C2.5t1.
